# The interactive effects of non-alcoholic fatty liver disease and hemoglobin concentration in the first trimester on the development of gestational diabetes mellitus

**DOI:** 10.1371/journal.pone.0257391

**Published:** 2021-09-13

**Authors:** Mengnan Li, Min Hu, Zhijing Yue, Yudan Zhang, Hailan Yang

**Affiliations:** 1 Department of Obstetrics, The First Hospital of Shanxi Medical University, Shanxi, China; 2 Department of Clinical Laboratory, The First Hospital of Shanxi Medical University, Shanxi, China; 3 Department of Ultrasonography, The First Hospital of Shanxi Medical University, Shanxi, China; University of Mississippi Medical Center, UNITED STATES

## Abstract

Gestational diabetes mellitus (GDM) is associated with adverse perinatal and maternal outcomes. Epidemiological studies have reported that non-alcoholic fatty liver disease (NAFLD) and a high hemoglobin (Hb) concentration are risk factors for GDM in the middle trimester. However, no consistent conclusions have been reached, especially in Chinese pregnant women. A case-control study was conducted to better understand the associations between NAFLD and Hb concentration in the first trimester and the risk of GDM and their interactive effects. Multivariable logistic regression analysis and a cross-product term of Hb and steatosis were used to evaluate the associations between first trimester Hb concentration, steatosis, and GDM and their interactive effects. Odds ratios (OR*s*) and 95% confidence intervals (CIs) were calculated using two-sided statistical tests at an alpha level of 0.05. For the study, 1,017 normal pregnant women, and 343 pregnant women diagnosed with GDM (25.22%) were recruited from the First Hospital of Shanxi Medical University, Shanxi Province, China. NAFLD-associated steatosis was found to be independent risk factors for developing GDM compared with grade 0 steatosis, with ORs of 1.98 (95% CI: 1.35–2.89) and 2.27 (95% CI:1.29–3.96), respectively. Meanwhile, a high Hb concentration was found to be a risk factor for developing GDM compared with the normal Hb concentration (OR = 1.88; 95% CI:1.24–2.83). The risk of developing GDM was more pronounced among pregnant women who had both high-grade steatosis and higher Hb concentrations during their first trimester (OR = 6.24; 95% *CI*: 1.81–23.66). However, we found no significant interactions between Hb concentration and steatosis grade. In conclusion, our study confirmed that a high Hb concentration and NAFLD-associated steatosis during the first trimester play important roles in predicting the risk of GDM in Chinese women. Future studies are required to verify the interactive effects between NAFLD-associated steatosis and Hb concentration.

## Introduction

Gestational diabetes mellitus (GDM) is defined as any degree of glucose intolerance with an onset, or first diagnosis, during pregnancy in women who did not have overt antenatal diabetes [1]. The incidence of GDM is increasing, especially in Asian woman, which has led to an increase in the service burden and health-care costs associated with pregnancy [2]. GDM is also associated with adverse perinatal outcomes (e.g, fetal macrosomia, preterm birth, birth trauma, and cesarean delivery) and maternal outcomes (e.g, type 2 DM and preeclampsia) [3,4]. Clinical practice guidelines recommend screening for GDM at 24–28 weeks of gestation [5]. However, the mid-pregnancy timeframe may be too late to favorably affect maternal and fetal outcomes. Thus, it is clinically important to identify risk factors for GDM to improve clinical outcomes for both mothers and infants and reduce the incidence of GDM by earlier intervention.

GDM is a complex multifactorial process that involves advanced maternal age, high body mass index (BMI) during pregnancy, obesity, and non-alcoholic fatty liver disease (NAFLD) [6,7]. A meta-analysis showed that women with GDM are at increased risk of developing NAFLD later in life [8]. However, the significance of NAFLD in women during their first trimester of pregnancy has not been fully studied. NAFLD is a major manifestation of the presence of insulin resistance (IR) and metabolic syndrome. It is noteworthy that IR and metabolic syndrome in pregnant women can lead to the development of GDM [9]. Epidemiological studies have reported that NAFLD is associated with an increased risk of impaired glucose tolerance in non-pregnant patients, and NAFLD in early pregnancy has been shown to be a risk factor for GDM in the middle trimester [7,10]. However, the relationship between NAFLD and IR appears to vary greatly according to the characteristics of the population [11]. Thus, it is important to demonstrate the reproducibility of these results in the Chinese population.

Obesity and high BMI are traditional risk factors for GDM, and previous studies have indicated that there is a significant association between hemoglobin (Hb) concentration during pregnancy and BMI [12]. In China, Hb concentration is routinely measured in pregnant women during their first perinatal visit. Doctors and pregnant women pay much attention to anemia, because it is a risk factor for preterm birth, low birth weight, infection, and maternal morbidity [13]. However, a high Hb concentration has not received the same attention as anemia. Lao et al. [14] first suggested that a high maternal Hb concentration (more than 13 g/dL) at the initial prenatal visit is an independent risk factor for GDM in Chinese women. However, inconsistent results have been reported regarding the Hb concentration and the risk of GDM [15,16].

Previous studies have demonstrated that serum Hb concentrations are significantly higher in patients with NAFLD than in healthy control populations [17,18], and a cross-sectional study showed that elevated concentrations of circulating Hb increase proportionally with the level of steatosis in patients with NAFLD in Mexico, which suggests that Hb acts as an antioxidant [19]. It is unclear whether the associations between NAFLD in the first trimester and GDM are modified by maternal Hb concentrations, and no study has evaluated these interactive effects.

In this study, we aimed to explore the association between NAFLD and the Hb concentration during the first prenatal care visit from 11 to 13 weeks of gestation and the subsequent risk of developing GDM in pregnant Chinese women. Moreover, we evaluated the potential interaction between NAFLD and Hb concentration in the development of GDM.

## Materials and methods

### Study design and population

This case-control study was conducted at the outpatient clinic of the Department of Obstetrics at the First Hospital of Shanxi Medical University, Shanxi Province, China. The cases were diagnosed as GDM at 24–28 weeks of gestation, and the controls were normal pregnant women (non-GDM). The pregnant women who met those following criteria were included in this study: (1) Residents of Taiyuan, aged 20 years or older, with singleton pregnancies; (2) presenting for prenatal care at the first prenatal care visit from 11 to 13 weeks of gestation from January 2018 to May 2019 and voluntarily participating to a separate protocol focused on screening for preeclampsia; (3) interviewed by local healthcare professionals through face-to-face using a brief structured questionnaire; (4) assessed for hepatic fat content by using liver ultrasonography; (5) received oral glucose tolerance test (OGTT) at 24–28 weeks of gestation. All electronic medical records of included pregnant women were systematically searched by information department.

Women were excluded from this study if they had previous diagnosis of GDM [20], had a family history of diabetes, had pregestational hypertension or other endocrine or metabolic disorders, or were taking medications that affect insulin or glucose levels. Women with hepatitis B, primary biliary cholangitis, or other chronic liver disease during pregnancy; a history of alcohol use or smoking; or incomplete OGTT results were also excluded.

The Ethics Committee of the First Hospital of Shanxi Medical University approved this study. The Information Department of the hospital integrated individual data from April 2, 2020 to May 10, 2020, and the final data with anonymized information were sent to our team for further analysis. As the data were anonymous, the requirement for informed consent was waived.

### The diagnosis of GDM

At 24–28 weeks of gestation, all pregnant women underwent a universal 75 g OGTT in the morning. Venous blood samples were drawn at 0, 1, and 2 hours after glucose loading. Women with a glucose concentration ≥ 5.1 mmol/L at 0 h, ≥ 10 mmol/L at 1 h, or ≥ 8.5 mmol/L at 2 h were diagnosed with GDM in accordance with the criteria recommended by the International Association of Diabetes and Pregnancy Study Group (IADPSG) [21].

### Hb concentration

Peripheral venous blood samples were drawn into EDTA-K2 anticoagulation tubes at the first prenatal care visit from 11 to 13 weeks of gestation. Hb concentration was measured with a Sysmex XN-1000 hematology analyzer (Sysmex Corporation, Kobe, Japan) using the sodium dodecyl sulfate Hb method. The recommended reference range for Hb concentration in pregnant women is 115–150 g/L when measured using the XN-1000 hematology analyzer. We used clinical cutoffs based on the reference concentrations to categorize subjects into the following three groups at the first trimester: low Hb group (< 115 g/L), normal Hb group (115–150 g/L), and high Hb group (≥ 150 g/L).

### Diagnosis of NAFLD based on liver ultrasonography

Pregnant women were assessed for hepatic fat content at their first prenatal care visit by liver ultrasonography using a Vivid S6 instrument (GE Healthcare, Chicago, IL, USA). The assessment was provided free of charge. According to the degree of hepatic steatosis [10,22], each woman was given a grade of 0, 1, 2, or 3 by two trained technicians. Any disagreement in the assessment of steatosis grade was resolved by another experienced sonographer. Because few patients were assessed as having grade 3 steatosis, we categorized participants into the following three groups: grade 0, grade 1, and grade 2 or 3 steatosis groups.

### Other covariates

A brief structured questionnaire was used by local healthcare professionals to collect potential confounders from each pregnant woman at their first prenatal care visit through face-to-face interviews. The demographic characteristics, such as maternal age, age of menarche, gestational week, parity, race, educational level, monthly income, and self-reported pre-pregnancy weight and height, were collected from the medical records. Body weight and height at 11–13 and 24–28 weeks of gestation were also measured at routine examinations using an Omron HNH-219 automate devices (Omron Healthcare, Kyoto, Japan). The women wore light clothing and no shoes during these measurements. Gestational age was calculated according to the last menstrual period, and BMI was calculated as weight (kg) divided by height (m^2^). The clinical characteristics, systolic blood pressure (SBP), diastolic blood pressure (DBP), fasting blood glucose (FBG) concentration, glycosylated hemoglobin (HbA1c) concentration, aspartate aminotransferase (AST) concentration, and alanine aminotransferase (ALT) concentration during the first trimesters (11–13 weeks) were also collected from medical records. Blood pressure was measured in the seated position using an automatic sphygmomanometer (HBP-9021, Omron) in the same arm in all patients. After overnight fasting, blood samples were drawn from the antecubital vein in the morning, and glucose oxidase (AU5800; Beckman Coulter, Inc., Brea, CA, USA) and latex agglutination methods (LABOSPECT 008AS; Hitachi, Tokyo, Japan) were used to determine FBG and HbA1c concentrations within 4 h after blood collection.

### Statistical analysis

Continuous variables were analyzed using the Kolmogorov Smirnov test to determine whether they were normally distributed. For normally distributed values, the mean ± standard deviation (SD) is presented, and Student’s t-test was used to compare the GDM and non-GDM groups. Otherwise, the Mann Whitney U test was used to compare parameters between groups, and values are presented as medians (P_25_, P_75_). The number and percentage are presented for categorical variables, and a chi-square (χ^2^) test was used to compare these parameters between the GDM and non-GDM groups. For ranked data, such as educational level, monthly income, first trimester Hb group, and steatosis grade, the Mann Whitney U test was used for comparisons between two groups. To assess the association between the first trimester Hb concentration and steatosis grade determined by liver ultrasonography and GDM at 24–28 weeks, a binary logistic regression model was generated.

Multivariate logistic regression analysis after adjustment for demographic and clinical confounding factors, was also used to evaluate the associations between first trimester Hb concentration and steatosis and GDM at 24–28 weeks. The confounders are presented in S1 Table in S1 Appendix. Because of the high correlations between the pre-pregnancy BMI, BMI at 11–13 weeks, and BMI at 24–28 weeks, we built three different multivariate models to adjust for BMI. To assess the model fit, the smallest Akaike information criterion (AIC) was used as the optimal goodness of fit statistic [23].

To explore the effect of the modification by Hb concentration on the association between steatosis and GDM, we stratified the models by first trimester Hb concentration. A cross-product term of Hb and steatosis was included in the models, and a *p*-value less than 0.05 for the interaction term was used to indicate a significant effect modification. Odds ratios (ORs), adjusted ORs *(*aOR), and 95% confidence intervals (CIs) were determined using two-sided statistical tests at an alpha level of 0.05. Data analyses were conducted using the statistical packages R (The R Foundation; http://www.r-project.org; version 4.0.2).

## Results

### Characteristics of the two groups

**Fig 1:**
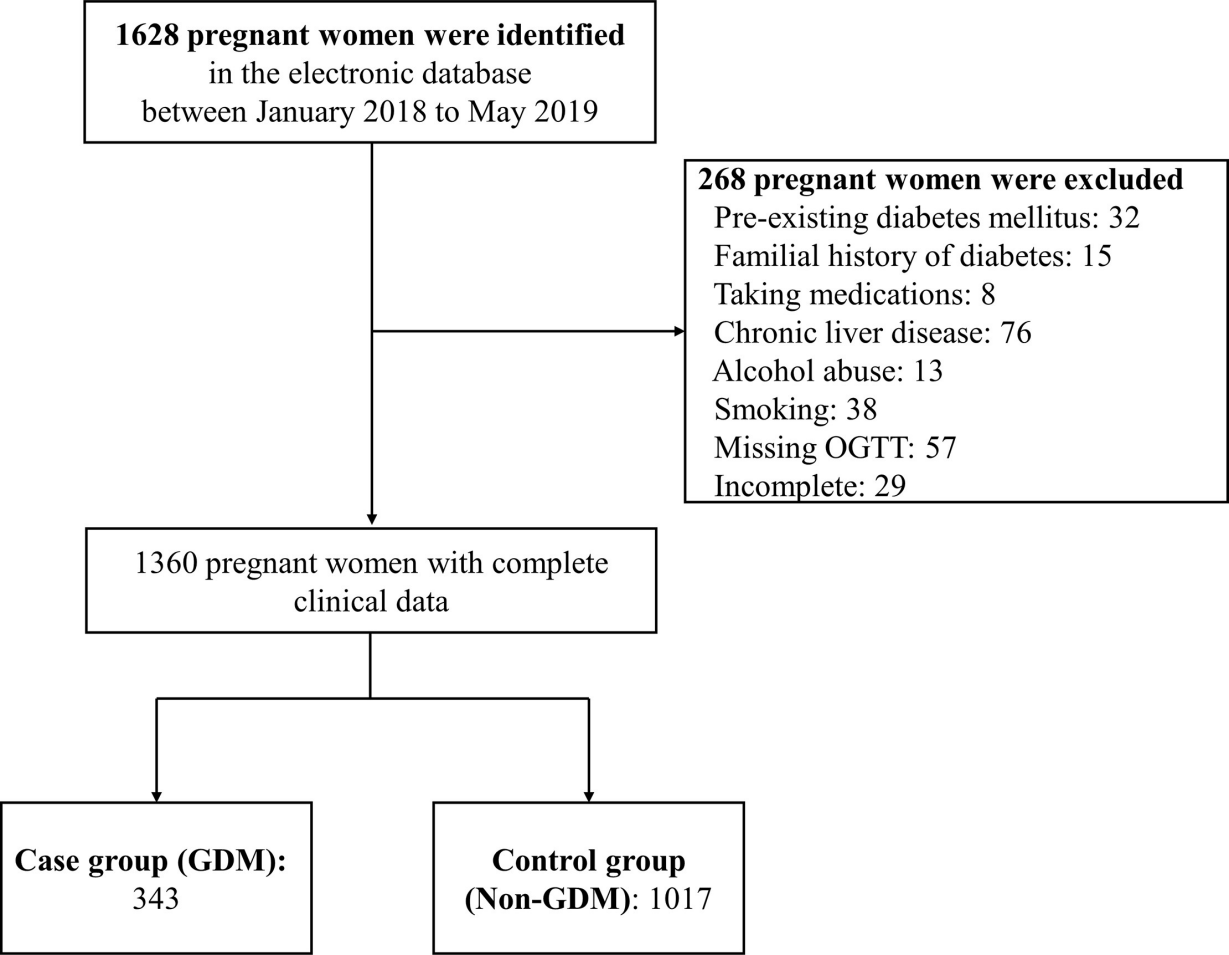
Flow chart of the study enrolment.

Of 1,628 pregnant women, 1,360 met the inclusion criteria for this study from January 2018 to May 2019, with a median age of 29 years and an average pre-pregnancy BMI of 20.91 (kg/m^2^). All participants were of Han Chinese ethnicity. Two hundred and sixty-eight women were excluded. A flow chart of the enrolment process is shown in Fig 1.

Three hundred and forty-three pregnant women were diagnosed with GDM (25.22%), and 1,017 had normal OGTT results at 24–28 weeks. The median maternal age, BMI at 24–28 weeks, FBG concentration, AST and ALT concentrations, mean HbA1c concentration, and percentage of multiparous pregnancies were all significantly higher in the GDM group than the non-GDM group (*p* < 0.05). The first trimester Hb concentration was significantly higher in women with GDM (130.96, g/L) than in women without GDM (126.11, g/L). Meanwhile, there were significant differences in the categories of high first trimester Hb concentration and steatosis grade between the two groups, with more pregnant women in the GDM group having a high Hb concentration (21.28%) and grade 2 or 3 steatosis (16.33%, Table 1).

**Table 1:** The demographic and clinical characteristics of pregnancy woman in this study.

Variables	Total (*n* = 1360)	Non-GDM (*n* = 1017)	GDM (*n* = 343)	*P* value
Age (years)	29(27, 32)	29(27, 32)	30(27.5, 33)	0.009
Pre-pregnancy BMI (kg/m^2^)	20.91 (18.98, 22.51)	20.71 (18.95, 22.28)	21.42 (19.20, 23.18)	0.090
Age of menarche (years)	13(12, 15)	13(12, 15)	14(12, 16)	0.790
Education				0.556*
Middle school below	366(26.91)	275(27.04)	91(26.53)	
High school	368(27.06)	266(26.16)	102(29.74)	
Bachelor or above	626(46.03)	476(46.80)	150(43.73)	
SBP (mmHg)	118(111, 125)	118(111, 125)	117(110, 124)	0.539
DBP (mmHg)	70(67, 75)	70(67, 75)	71(67, 75.5)	0.818
Monthly income (RMB)				0.802*
<2000	359(26.40)	266(26.16)	93(27.11)	
2000–3500	486(35.74)	365(35.89)	121(35.28)	
≥3500	515(37.87)	386(37.95)	129(37.61)	
BMI at 11–13 weeks (kg/m^2^)	23.14 (20.02, 26.05)	23.01 (19.97, 25.70)	23.64 (20.23, 27.11)	0.278
BMI at 24–28 weeks (kg/m^2^)	28.52 (25.02, 32.22)	27.96 (24.72, 31.40)	29.93 (25.96, 33.98)	0.010
Parity				0.044
Primiparous	1002(73.68)	764(75.12)	238(69.39)	
Multiparous	358(26.32)	253(24.88)	105(30.61)	
First trimester Hb (g/L)	126.86 (117.53, 134.04)	126.11 (114.43, 133.29)	130.96 (119.74, 135.60)	<0.001
<115	321(23.60)	257(25.27)	64(18.66)	0.020*
115–150	856(62.94)	650(63.91)	206(60.06)	
≥150	183(13.46)	110(10.82)	73(21.28)	
Steatosis				<0.001*
Grade 0	991(72.87)	782(76.89)	209(60.93)	
Grade 1	239(17.57)	161(15.83)	78(22.74)	
Grade 2 or 3	130(9.56)	74(7.28)	56(16.33)	
HbA1c (%)	5.57±0.33	5.51±0.30	5.76±0.34	<0.01
FBG (mmol/L)	4.50(4.41, 4.60)	4.48(4.39, 4.56)	4.63(4.49, 4.78)	0.001
AST/ALT	1.24(1.15, 1.35)	1.23(1.15, 1.32)	1.28(1.14, 1.43)	<0.001

BMI, body mass index; GDM, gestational diabetes mellitus; *P*-value is probability value of hypothesis test of differences between GDM and non-GDM; SBP, Systolic blood pressure; DBP, Diastolic blood pressure; Hb, hemoglobin; HbA1c, Glycosylated hemoglobin; FBG, Fasting blood glucose; AST: Aspartate aminotransferase; ALT: Alanine aminotransferase.

* Mann Whitney U test was used for *P* value.

### Associations between confounders and GDM risks

We first performed a univariate analysis of all variables, except Hb concentration and steatosis grade, to assess their effects on the risk of developing GDM. The results of the univariate analysis are shown in S2 Table in S1 Appendix. We found that increased maternal age; a high pre-pregnancy BMI; high BMI at 10–14 and 24–28 weeks; higher parity; and high HbA1c, FBG, AST, and ALT concentrations were all risk factors for GDM.

Compared with women in the normal Hb group, pregnant women in the low Hb group did not have an increased risk of developing GDM. However, a significantly higher OR for developing GDM was observed among pregnant women in the high Hb group (unadjusted OR = 2.09, 95% CI: 1.49–2.92). Because of the high correlations between pre-pregnancy BMI, BMI at 11–13 weeks, and BMI at 24–28 weeks, we built models 2, 3, and 4, respectively, to adjust for these parameters. The results remained robust even after adjusting for covariates (Table 2). The risk of GDM in the high Hb group was attenuated in models 2, 3, and 4 (Table 2).

**Table 2:** OR and 95% CI in univariate and multivariate analysis of steatosis and Hb concentration during the first trimester for developing GDM.

Variables	Model 1^a^	Model 2^b^	Model 3^c^	Model 4^d^
First trimester Hb (g/L)				
115~150	Ref	Ref	Ref	Ref
<115	0.79 (0.57–1.07)	0.81 (0.55–1.17)	0.81 (0.55–1.17)	0.77 (0.52–1.12)
≥150	2.09 (1.49–2.92)	1.92 (1.27–2.88)	1.87 (1.24–2.82)	1.88 (1.24–2.83)
AIC	1517.60	1125.70	1128.76	1112.69
Steatosis				
Grade 0	Ref	Ref	Ref	Ref
Grade 1	1.81 (1.32–2.47)	2.02 (1.39–2.94)	2.03 (1.40–2.95)	1.98 (1.35–2.89)
Grade 2 or 3	2.83 (1.93–4.13)	2.25 (1.28–3.93)	2.19 (1.25–3.80)	2.27 (1.29–3.96)
AIC	1506.62	1120.88	1123.24	1108.71

OR, odds ratio; CI, confidence interval; Hb, hemoglobin; GDM, gestational diabetes mellitus; AIC, Akaike Information Criterion; BMI, body mass index; HbA1c, Glycosylated hemoglobin; FBG, Fasting blood glucose.

^a^ Model 1: Unadjusted.

^b^ Model 2: Adjusting for age; BMI at 24–28 weeks; parity; and HbA1c, FBG, AST, and ALT concentrations.

^c^ Model 3: Adjusting for age; BMI at 24–28 weeks; parity; and HbA1c, FBG, AST, and ALT concentrations.

^d^ Model 4: Adjusting for age; BMI at 24–28 weeks; parity; and HbA1c, FBG, AST, and ALT concentrations.

Compared with grade 0 steatosis, as determined by liver ultrasonography, pregnant women with grade 1 steatosis had a higher risk of developing GDM in the unadjusted model (OR = 1.81, 95% *CI*: 1.32–2.47). Moreover, a significantly higher *OR* of developing GDM (unadjusted OR = 2.83, 95% *CI*: 1.93–4.13) was also observed among pregnant women with grade 2 or 3 steatosis, even after adjusting for other covariates (Table 2).

### Interactive effects between steatosis and Hb concentration

Among pregnant women in the low Hb group (< 115 g/L), the OR increased with increasing steatosis grade, with a significant association for grade 2 or 3 vs. grade 0 (unadjusted OR = 2.99, 95% *CI*: 1.28–6.7, *p*-trend = 0.004). As shown in Table 2, the lowest AIC values were all found in model 4; thus, BMI at 24–28 weeks was the factor with the greatest influence on the risk of GDM, compared with pre-pregnancy BMI and BMI at 11–13 weeks. However, this association attenuated to non-significant after adjusting for other covariates in model 2 (aOR = 2.68, 95% *CI*: 0.87–8.26; Table 3).

**Table 3:** OR and 95% CI between steatosis and GDM stratified by first trimester Hb concentration.

Variables	GDM/Total	Model 1^a^	Model 2^b^
Lower of Hb			
Grade 0	38/234	Ref	Ref
Grade 1	15/57	1.84 (0.91–3.60)	1.76 (0.70–4.31)
Grade 2 or 3	11//30	2.99 (1.28–6.70)	2.68 (0.87–8.26)
*P* value for trend	-	0.004	0.027
Normal of Hb			
Grade 0	135/637	Ref	Ref
Grade 1	41/141	1.52 (1.00–2.28)	1.80 (1.09–2.28)
Grade 2 or 3	30/78	2.32 (1.41–3.79)	2.41 (1.19–4.88)
*P* value for trend	-	<0.001	<0.001
Higher of Hb			
Grade 0	36/120	Ref	Ref
Grade 1	22/41	2.70 (1.31–5.64)	2.79 (1.16–6.90)
Grade 2 or 3	15/22	5.00 (1.94–14.08)	6.24 (1.81–23.66)
*P* value for trend	-	<0.001	<0.001
*P* value for interaction		0.392	0.058

OR, odds ratio; CI, confidence interval; Hb, hemoglobin; GDM, gestational diabetes mellitus; BMI, body mass index; HbA1c, Glycosylated hemoglobin; FBG, Fasting blood glucose.

^a^ Model 1: Unadjusted.

^b^ Model 2: Adjusting for age; BMI at 24–28 weeks; parity; and HbA1c, FBG, AST, and ALT concentrations.

Among pregnant women in the normal Hb group (115–150 g/L), the risk of GDM increased with increasing steatosis grade in models 1 and 2 (*p*-trend < 0.001). The ORs increased after adjusting for age; BMI at 24–28 weeks; parity; and HbA1c, FBG, AST, and ALT concentrations, with significant associations for grade 1 vs. grade 0 steatosis (aOR = 1.80, 95% *CI*: 1.09–2.28) and grade 2 or 3 vs. grade 0 steatosis (aOR = 2.41, 95% *CI*: 1.19–4.88; Table 3).

The same results were observed for pregnant women in the high Hb group (> 150 g/L, *p*-trend < 0.001). The ORs increased after adjusting for age; BMI at 24–28 weeks; parity; and HbA1c and FBG concentrations, with significant associations for grade 1 vs. grade 0 steatosis (aOR = 2.79, 95% CI: 1.16–6.90), and grade 2 or 3 vs. grade 0 steatosis (aOR = 6.24, 95% CI: 1.81–23.66; Table 3). Although the risk of GDM increased with increasing Hb concentration, the interaction between Hb concentration and steatosis grade was not significant in model 1 (*p* = 0.392) or model 2 (*p* = 0.058).

## Discussion

To the best of our knowledge, this is the first study to clearly demonstrate an interaction between NAFLD-associated steatosis and Hb concentration in determining the risk of GDM in pregnant Chinese women. We found that NAFLD-associated steatosis and a high Hb concentration in the first trimester were independent risk factors for developing GDM after adjusting for confounding factors. In addition, when we stratified by Hb concentration, the joint effects on the risk of GDM were more pronounced among pregnant women who had high-grade steatosis with higher Hb concentration during their first trimester (OR = 6.24, 95% CI: 1.81–23.66). However, we did not find significant interaction effects between Hb concentration and steatosis grade.

GDM is one of the most common complications affecting pregnant woman, with an incidence varying from 2.9% to 32.1% [24,25]. Based on 1999 World Health Organization or IADPSG criteria, the prevalence of GDM in China is approximately 17% [26], and its incidence is rapidly increasing due to the increasing prevalence of obesity and type 2 DM. In this study, the incidence of GDM was 25.22%, which was higher than previous reports [27]. The reasons for this discrepancy may be the wide variation in study designs, population characteristics, sample sizes, and the time of GDM onset.

The known risk factors for GDM include advanced maternal age, a history of type 2 diabetes, a history of GDM, maternal overweight and obesity, and race/ethnicity [28]. In this study, we excluded those who had a previous diagnosis of GDM or a familial history of diabetes, which may lead to diabetes-related changes in NAFLD and glucose concentration. In our study, we found that age, high pre-pregnancy BMI, high BMI at 11–13 weeks, high BMI at 24–28 weeks, high HbA1c and FBG concentrations, and the percentage of multiparous pregnancies were all significantly higher in the GDM group than the non-GDM group. Our findings are generally in line with those of other studies [27,29].

We first demonstrated that the presence of NAFLD (grade 1 and above) in the early pregnancy was associated with the subsequent development of GDM in a Chinese population, and this association persisted even after adjusting for age, BMI at 24–28 weeks, parity, and HbA1c and FBG concentrations. A previous meta-analysis showed that Western women with NAFLD are at increased risk of developing GDM during pregnancy [30]. The same result was reported in a Korean population [10], which is consistent with our findings. Liver ultrasound is a safe, a simple and standardized imaging modality for the assessment of hepatic fat levels, which can be used to predict GDM. NAFLD may cause impaired glucose tolerance and increase the risk of developing GDM; however, the associated mechanism is not clear. NAFLD and GDM are metabolic diseases that share a common metabolic dysfunction, namely IR [9]. An animal study reported that increased hepatic activating transcription factor 3 levels play an important role in hepatic steatosis, IR, and subsequent type 2 DM [31].

Hb measurement is a simple and standard test to evaluate physical status and anemia in pregnant women during their first trimester. Our results demonstrated that women with Hb concentrations ≥150 g/L had an increased risk of GDM during early pregnancy in a Chinese population, and this association persisted even after adjusting for age, BMI at 24–28 weeks, parity, and HbA1c and FBG concentrations. Similar to our results, a hospital-based retrospective study of 21,577 pregnant Chinese women reported that high maternal Hb concentrations (≥150 g/L) in the first trimester are associated with a 1.27-fold increased risk of developing GDM [32]. Another hospital-based cohort study conducted in Hangzhou, also revealed that high Hb concentrations and iron supplementation increase the risk of GDM [33]. However, Zhu et al. [27] did not find a significant association between Hb concentration and GDM after adjusting for pre-pregnancy BMI and blood pressure.

These findings are inconsistent mainly due to variations in study designs (cohort, case-control study), sample sizes, study regions, and the time of Hb testing. Furthermore, we did not find significant associations between a low Hb concentration in the first trimester and GDM in the mid-pregnancy phase in the four models. Generally, when anemia occurs during pregnancy, doctors recommend iron supplementation to prevent poor neonatal outcomes. Thus, women with low Hb concentration in the first trimester would have normal Hb concentrations after taking iron supplements.

We also explored the effect of Hb concentration on the association between liver steatosis and the risk of GDM. Importantly, we determined the thresholds for Hb concentration (≥150 g/L) and hepatic steatosis during the first trimester that identify pregnant women with a high risk of GDM in China, especially among those with grade 2 or 3 steatosis. Among pregnant women in the low Hb group (< 115 g/L), there was no significant association between steatosis grade and GDM (aOR = 2.68, 95% CI: 0.87–8.26) after including other covariates in the analysis. As mentioned above, once anemia occurs during pregnancy, women are more likely to take iron supplements to maintain their Hb concentration. Thus, early testing, intervention, and disease-modifying lifestyle changes may effectively prevent GDM. Among pregnant women in the normal Hb group (115–150 g/L), the risk of GDM increased with increasing steatosis grade, even after including other covariates in the analysis, which indicated that NAFLD plays an important role in determining the risk of GDM. Meanwhile, the risk of developing GDM was more pronounced among pregnant women who had high-grade steatosis with higher Hb concentration during their first trimester. However, we did not find an interaction effect in this study, which may due to the small number of patients in each stratum of Hb concentration and steatosis grade. Moreover, the CIs around the ORs were wide, which may suggest a lack of power. Thus, further studies should be performed to confirm this interactive effect.

There were some limitations of this study that should be taken into account. First, our study subjects were recruited at a single hospital; thus, the results should be interpreted with caution when generalizing to other populations. Multicenter studies should be performed to further confirm the association. Second, liver ultrasound is a semi-quantitative method of assessing hepatic fat content and may only yield positive results if the hepatic fat content exceeds 30% [34]. Thus, minor hepatic steatosis may not be detected. Additionally, there was a lack of information about genetic liver disease, and therefore, some pregnant women with autoimmune or genetic liver disease may not have excluded. However, the proportion of these cases was small, and thus they are not likely to have significantly biased the results. Third, we only measured steatosis and Hb concentrations in the first trimester. As these are dynamic metrics associated with weight gain during pregnancy, their measurement in the second or third trimester would enhance our understanding of the link between NAFLD, Hb concentration, and GDM.

## Conclusions

Our study confirmed that a high Hb concentration and liver steatosis during the first trimester play important roles in predicting the risk of GDM in Chinese women, especially in pregnant women with high-grade steatosis and higher Hb concentrations. These findings are of clinical and public health importance, and they highlight the potential benefits of identifying women at high risk of GDM. Multicenter studies should be performed to further confirm these associations and assess their generalizability to other populations.

## Supporting information

S1 Appendix (DOCX)

S1 File (PDF)

S2 File (DOCX)

S3 File (PDF)

S4 File (PDF)

S5 File (DOC)
