# Supplementary material for: The interactive effects of non-alcoholic fatty liver disease and hemoglobin concentration in the first trimester on the development of gestational diabetes mellitus
Source: PLoS One. 2021 Sep 13;16(9):e0257391. doi: 10.1371/journal.pone.0257391 (PMC8437282; doi:10.1371/journal.pone.0257391)
Supplement: S1 Appendix — (DOCX) [file pone.0257391.s001.docx]

**S1 Table. The characteristics and cut-off values of confounders modelled in the regression models**

| **Variables** | **Type** | **Definition** |
| --- | --- | --- |
| Age | Continuous | years |
| Pre-pregnancy BMI | Continuous | kg/m^2^ |
| Age of menarche | Continuous | years |
| Education | Ranked data | 1=Middle school below; 2=High school; 3=bachelor or above |
| SBP | Continuous | mmHg |
| DBP | Continuous | mmHg |
| Monthly income | Ranked data | 1=<2000; 2=2000-3500; 3=≥3500 |
| BMI at 11-13 weeks | Continuous | kg/m^2^ |
| BMI at 24-28 weeks | Continuous | kg/m^2^ |
| Parity | Category | 0=Primiparous; 1=Multiparous |
| First trimester Hb (g/L) | Ranked data | 1 =<115; 2= 115-150; 3=≥150 |
| Steatosis | Ranked data | 1=Grade 0; 2=Grade 1; 3=Grade 2 or 3 |
| HbA1c | Continuous | % |
| FBG | Continuous | mmol/L |
| AST/ALT | Continuous |  |

BMI, body mass index; GDM, gestational diabetes mellitus; *P*-value is probability value of hypothesis test of differences between GDM and non-GDM; SBP, Systolic blood pressure; DBP, Diastolic blood pressure; Hb, hemoglobin; HbA1c, Glycosylated hemoglobin; FBG, Fasting blood glucose; AST: Aspartate aminotransferase; ALT: Alanine aminotransferase.

**S2 Table. *OR* and 95% *CI* in univariate analyses of confounders for GDM risk**

| **Variables** | **Estimate** | **Std. Error** | **Z value** | ***P* value** | ***OR* (95% *CI*)** |
| --- | --- | --- | --- | --- | --- |
| Age(years) | 0.078 | 0.015 | 5.096 | 0.000 | 1.08(1.05-1.11) |
| Pre-pregnancy BMI (kg/m^2^) | 0.098 | 0.027 | 3.707 | 0.000 | 1.10(1.05-1.16) |
| Age of menarche (years) | 0.032 | 0.031 | 1.034 | 0.301 | 1.03(0.97-1.10) |
| Education |  |  |  |  |  |
| Middle school below | Ref |  |  |  |  |
| High school | 0.147 | 0.168 | 0.878 | 0.380 | 1.16(0.83-1.61) |
| bachelor or above | -0.049 | 0.153 | -0.320 | 0.749 | 0.95(0.71-1.29) |
| Smoking | -0.184 | 0.299 | -0.615 | 0.538 | 0.83(0.45-1.46) |
| Drinking | -0.433 | 0.293 | -1.480 | 0.139 | 0.65(0.35-1.12) |
| SBP (mmHg) | -0.010 | 0.006 | -1.590 | 0.112 | 0.99(0.98-1.00) |
| DBP (mmHg) | 0.007 | 0.010 | 0.715 | 0.475 | 1.01(0.99-1.03) |
| Monthly income (RMB) |  |  |  |  |  |
| <2000 | Ref |  |  |  |  |
| 2000-3500 | -0.053 | 0.160 | -0.333 | 0.739 | 0.95(0.69-1.3) |
| ≥3500 | -0.045 | 0.158 | -0.286 | 0.775 | 0.96(0.7-1.3) |
| BMI at 10-14 weeks (kg/m^2^) | 0.051 | 0.017 | 2.953 | 0.003 | 1.05(1.02-1.09) |
| BMI at 24-28 weeks (kg/m^2^) | 0.084 | 0.014 | 6.155 | 0.000 | 1.09(1.06-1.12) |
| Parity | 0.287 | 0.138 | 2.082 | 0.037 | 1.33(1.01-1.74) |
| HbA1c (%) | 0.084 | 0.029 | 2.866 | 0.004 | 1.09(1.03-1.15) |
| FBG (mmol/L) | 0.043 | 0.019 | 2.248 | 0.025 | 1.04(1.01-1.08) |
| AST/ALT | 0.146 | 0.048 | 3.042 | 0.002 | 1.16(1.05-1.27) |

OR, odds ratio; CI, confidence interval; BMI, body mass index; GDM, gestational diabetes mellitus; *P*-value is probability value of hypothesis test of differences between GDM and non-GDM; SBP, Systolic blood pressure; DBP, Diastolic blood pressure; HbA1c, Glycosylated hemoglobin; FBG, Fasting blood glucose; AST: Aspartate aminotransferase; ALT: Alanine aminotransferase.
