# Supplementary material for: The interactive effects of non-alcoholic fatty liver disease and hemoglobin concentration in the first trimester on the development of gestational diabetes mellitus
Source: PLoS One. 2021 Sep 13;16(9):e0257391. doi: 10.1371/journal.pone.0257391 (PMC8437282; doi:10.1371/journal.pone.0257391)
Supplement: S2 File — (DOCX) [file pone.0257391.s003.docx]

**Application Form for Medical Ethical Review of scientific research projects of First hospital of Shanxi medical university**

| **Certificate number** | | |  | | | **Organization** | | First hospital of Shanxi medical university | | |
| --- | --- | --- | --- | --- | --- | --- | --- | --- | --- | --- |
| **Project name** | The interactive effects of Non-alcoholic fatty liver disease and hemoglobin levels in the first trimester for developing gestational diabetes mellitus | | | | | | | [**Research**](D:/program_files/Youdao/Dict/8.9.3.0/resultui/html/index.html#/javascript:;)  [**period**](D:/program_files/Youdao/Dict/8.9.3.0/resultui/html/index.html#/javascript:;) | | 2020.1-2020.12 |
| **Type of project** | **A.** Clinical trials of new drugs **B.** Clinical trials of new device  **C.** [Application](javascript:;) [of](javascript:;) [new](javascript:;) [technology](javascript:;)  **D.** Collection of human specimens  **E.** Others：Retrospective cohort study | | | | | | | | | |
| **Summary of the Project** | | | | | | | | | | |
| **Name** | Hailan Yang | **Sex** | | female | **Education** | | Doctor | | **Department** | Obstetrics |
| **Office phone** | 0351-4639625 | **Fax** | |  | **Mobile phone** | | 13834048059 | | **E-mail** | lili-5y208@163.com |
| **Abstract of Ethical Review:**  **Objective:** To explore the association between non-alcoholic fatty liver disease (NAFLD) and hemoglobin (Hb) level in the first trimester and the risk of gestational diabetes mellitus (GDM) in Chinese pregnant woman.  **Methods:** A retrospective cohort study was conducted. Hepatic fat was assessed by using liver ultrasonography, and GDM diagnosis was tested with 75 g oral glucose tolerance test at 24-28 weeks of gestation. Multivariable logistic regression analysis was used to evaluate the associations between first trimester Hb and steatosis and GDM.  **Results:** Of 1017 woman, 343 pregnant women were diagnosed as GDM (25.22%). We found that the NAFLD steatosis and high Hb level in the first trimester were independent risk factors for developing GDM after adjusting for confounding factors. In addition, the joint effects of developing GDM were more pronounced among pregnant women who had high grade steatosis with higher Hb levels during their first trimester exposure with OR of 6.32 (95% CI: 2.14-20.41). However, we did not find the significant interactions between Hb level and steatosis grade.  **Conclusion:** Our study confirmed that high Hb levels and liver steatosis during the first trimester play an important role in predicting the risk of GDM in Chinese woman. | | | | | | | | | | |
| **A statement of applicant (project leader):**  All the above contents (including the attached materials) are true. If approved, I will carry out the research in strict accordance with the scheme provided and abide by the relevant regulations of the Medical Ethics Committee of the First Hospital of Shanxi Medical University.  Signature: Hailan Yang Date: 2019.12.15 | | | | | | | | | | |
| T**he opinions of the department:**  Agreement.  Signature: Hailan Yang Date: 2019.12.15 | | | | | | | | | | |
| **The opinions of the Ethics Committee members:**  It was discussed and approved by the ethics committee.    Signature: Qinghua Han Date: 2020.1.17 | | | | | | | | | | |
