# Supplementary material for: The interactive effects of non-alcoholic fatty liver disease and hemoglobin concentration in the first trimester on the development of gestational diabetes mellitus
Source: PLoS One. 2021 Sep 13;16(9):e0257391. doi: 10.1371/journal.pone.0257391 (PMC8437282; doi:10.1371/journal.pone.0257391)
Supplement: S3 File — (PDF) [file pone.0257391.s004.pdf]

山西省应用基础研究计划

项目申请书

项目名称：山西省子痫前期筛查质量控制系统研究项目

所属专项：面上项目

产业集群：妇产科学

研究方向：围产医学

项目起止时间：2018 年 01 月 01 日 至 2020 年 12 月 31 日

归口管理单位：山西省卫生健康委员会

申报单位：山西医科大学附属第一医院 （公章）

通讯地址：山西省太原市解放南路 85 号

邮政编码：030001

单位电话：0351-4639625

项目负责人：杨海澜

联系电话：13834048059

联系邮箱：lili-5y208@163.com

山西省卫生健康委员会

二〇一八年制

研究方案摘要

题目：	山西省子痫前期筛查质量控制系统研究项目
主要研究目的：	本研究依托国际妇产科联盟（FIGO）《孕早期子痫前期筛查和预防实用指南》及英国母胎医学基金会（FMF）给予贝叶斯法则制定的子痫前期筛查模型，结合山西地区实际情况，项目旨在加强山西省产前筛查机构的规范管理，优化子痫前期筛查工作流程，提高筛查医务人员的质量控制意识，从而切实提升相关医疗机构子痫前期筛查工作质量和技术水平，降低山西地区子痫前期发病率及死亡率，为我省妇幼保健事业发展贡献力量。<br>1、开展山西省孕妇子痫前期高风险因素人群的登记。<br>2、在 11-13 周$^{+6}$进行孕妇的危险因素调查、肝脏超声和实验室检测、平均动脉压(MAP)、血清胎盘生长因子(PLGF)及子宫动脉搏动指数(UTPI)的联合风险筛查，建立山西地区孕妇人群 MAP、PLGF、 UTPI 的本地化中位数值，提高筛查效率。
次要研究目的：	比较在真实的医疗环境下，山西太原地区孕产妇中与子痫前期预测模型相关的各个指标（MAP、PLGF、 UTPI 等）的中位数差异，及预测模型的临床截断值，并观察比较不同特征孕妇风险因素，不同监测时间、不同预防用药时间、不同停药时间对最终妊娠结局的差异，循证并制定符合山西地区的子痫前期风险评估与管理流程。<br>1、 结合孕早期、孕中期、孕晚期对孕妇母体相关危险因素调查和检测、MAP、PLGF、UTPI 组合的筛查，在真实环境下评价对早发型、晚发型、早产型不同截断值的筛查效率。<br>2、 通过科学筛查管理对孕产妇妊高症和子痫前期发病率及优生优育的影响。
其他研究目的：	1、探索医疗新技术，特别是妇幼健康相关的新技术在我国基层医疗机构的推广难点及解决方案。<br>2、探索 MAP、PLGF、UTPI 指标对胎盘功能引起妊娠疾病的评估方案。<br>3、作为远期观察研究，在有条件的情况下对孕产妇及新生儿未来生长发育的影响。

研究设计：	本次研究为登记人群队列、前瞻性、观察性研究
观察人群：	1、 入选标准：（1）常住居民（本地居住时间≥6月），孕期年龄≥20岁；（2）接受随访；（3）自愿参加筛查，并签署知情同意书。<br>2、 排除标准：（1）严重精神障碍，无法表达意愿者；（2）存在明显的其他异常体征、实验室检查和和临床疾病，根据研究者的判断，不适合参加研究者；（3）研究者判断无法完成长期随访者。
治疗方案：	本研究为观察性研究，不预先设立任何治疗方案，治疗方案完全由临床医生依据患者病情制定，本研究不做任何干预。
样本量：	≥3000例
观察时间：	3年
安全性考察：	根据国家药监局规定收集和报告不良事件。
统计分析内容：	1、 比较子痫前期孕妇与非子痫前期孕妇在怀孕过程、PIGF、MAP、UTPI等检测指标的人群分布差异。<br>2、评价在不同截断值下的预测模型与最后发生子痫前期的准确性。<br>3、分析不同母体情况及产检时发现其他临床疑似症状时与未来子痫前期发病率的相关性及纳入子痫前期风险评估与管理体系对提高子痫前期诊断，降低发病率等方面的获益。<br>4、分析通过全省推广科学的子痫前期风险评估与管理模式后，本地区孕产妇子痫前期发病率、死亡率、新生儿早产率的下降等方面的影响。
