# Supplementary material for: The interactive effects of non-alcoholic fatty liver disease and hemoglobin concentration in the first trimester on the development of gestational diabetes mellitus
Source: PLoS One. 2021 Sep 13;16(9):e0257391. doi: 10.1371/journal.pone.0257391 (PMC8437282; doi:10.1371/journal.pone.0257391)
Supplement: S4 File — (PDF) [file pone.0257391.s005.pdf]

Local Knowledge, Global Talent

Armstrong-Hilton Ltd.
2101 Bayfield Building, 99 Hennessy Road, Wanchai, Hong Kong
Tel: +852 2590-6588
Fax: +852 2856-0735
Web: www.asiaedit.com

Certificate

Certificate Date: 13/08/2021
Reference: AE-33405
Customer Id: 14068

To Whom it May Concern

This is to certify that the document listed below has been edited for English language by the professional academic editing company Armstrong-Hilton Limited.

- Document Name: The interactive effects of Non-alcoholic fatty liver disease and hemoglobin levels in the first trimester for developing gestational diabetes mellitus
- Author(s): Hainan Yang
- Date: 13/08/2021
- Reference: AE-33405

If you have any questions please contact us at cs@asiaedit.com quoting the reference number above.

Armstrong-Hilton has been editing and copywriting for the individuals, departments, schools and faculties of Asia’s leading academic institutions for 21 years. For more details please see www.asiaedit.com.

For and on behalf of
Armstrong-Hilton Ltd.

ARMSTRONG-HILTON HONG KONG EDITING

Armstrong-Hilton Ltd. (Authorized Chop)
