# Supplementary material for: The interactive effects of non-alcoholic fatty liver disease and hemoglobin concentration in the first trimester on the development of gestational diabetes mellitus
Source: PLoS One. 2021 Sep 13;16(9):e0257391. doi: 10.1371/journal.pone.0257391 (PMC8437282; doi:10.1371/journal.pone.0257391)
Supplement: S5 File — (DOC) [file pone.0257391.s006.doc]

**The protocol for Applied basic Research Program of Shanxi Province**

**Project name:** The quality control system research of screening for preeclampsia in Shanxi province

**Research direction:**  Perinatal medicine

**Funding category:**  Project approval

**Research time:**  From January 1, 2018 to December 31, 2020

**Management institution (first party):** Health commission of Shanxi province

**Research institution (second party):**  The first hospital of Shanxi medical University

**Address:**  85 Jiefang Road, Taiyuan City, Shanxi Province, China

**Postal code:**  030001

**Business phone No:**  0351-4639625

**Person in charge:** Hainan Yang

**Telephone**: 13834048059

**Email:**  [lili-5y208@163.com](mailto:lili-5y208@163.com)

**Health commission of Shanxi province**

**The year of 2018**

**Abstract for protocol**

| **Title** | The quality control system research of screening for preeclampsia in Shanxi province |
| --- | --- |
| **The primary objective** | This study was based on the International Federation of Gynecology and Obstetrics (FIGO) initiative on preeclampsia: A pragmatic guide for first-trimester screening and prevention and screening for preeclampsia model according to bayesian rule by the Fetal Medicine Foundation (FMF). Considering the practical situation of Shanxi province, we aim to optimize of screening process for preeclampsia, improve the quality control consciousness of screening of medical staff, and so as to effectively improve the quality and technical level of preeclampsia screening in relevant medical institutions, and to reduce the incidence and mortality of preeclampsia in Shanxi province. This research would contribute to the development of maternal and child health care in Shanxi province.   1. To register pregnant women with preeclampsia risk factors in Shanxi province. 2. The investigation for risk factors, liver ultrasonography, clinical tests, mean arterial pressure (MAP), placental growth factor (PLGF), and uterine artery pulsatility index (UTPI) would be measured for pregnant women at 11-13(+ 6) weeks' gestation. We also will construct the local pregnant median values of MAP, PLGF and UTPI for Shanxi province, so as to improve the screening efficiency. |
| **The second objective** | In the real medical world, we will compare the median values of each index (MAP, PLGF, UTPI, and etc.), which related to the prediction model of preeclampsia among pregnant women in Taiyuan city of Shanxi province, and we also compare the clinical cutoff values of the prediction model. We will compare the risk factors of pregnant women with different characteristics, and difference of the final pregnancy outcome under different monitoring time, different time of prophylactic medication and different time of drug withdrawal. At last, we will conduct the evidence-based preeclampsia risk assessment and management process in accordance with Shanxi province.   1. In the real medical world, screening efficiency of different clinical cutoff values will be evaluated under early-onset, late-onset and premature preeclampsia using risk factors, liver ultrasonography, clinical tests, MAP, PLGF, and UTPI of pregnant women at first trimester, second trimester, and third trimester. 2. To influence the incidence of pregnancy-induced hypertension and preeclampsia, as well as the bear and rear better children according to the scientific screening and management process. |
| **Other objectives** | 1. To explore the difficulties and solutions of the promotion of new medical technologies, especially the new technologies related to maternal and child health, in China's primary medical institutions.  2. To explore the evaluation of MAP, PLGF and UTPI for pregnancy diseases caused by placental function.  3. To observe the long-term influence of pregnant women and the growth and development of newborns, in the case of conditions that there are plenty of time and funds. |
| **Study design** | This study is a prospective, observational, population-based cohort study. |
| **Population** | Inclusion criteria: (1) Resident (local resident ≥6 months) and pregnant age≥20 years old; (2) Accept to follow-up; (3) Voluntary participation and signed an informed consent form before participating.  Exclusion criteria: (1) Severe mental disorder and unable to express their willness; (2) The presence of significant other abnormal signs, laboratory tests, and disease, which is not suitable to participate in the research judged by the researcher; (3) Pregnant women who is impossible to complete the long-term interview judged by the researcher. |
| **Treatment** | This study is an observational study with no intervention. The treatment plan is completely formulated by clinicians according to the patient's condition. |
| **Sample size** | ≥ 3000 |
| **Time** | Three years |
| **Safety** | Collect and report adverse events according to FDA regulations. |
| **Statistical analysis** | 1. Compare the population distribution in the process of pregnancy, PIGF, MAP, UTPI and other clinical test between preeclampsia pregnant women and non-preeclampsia pregnant women. 2. To verify the accuracy of the prediction models for occurrence of preeclampsia under different cutoff values. 3. To analyze the correlation between different maternal conditions and other clinical suspected symptoms and the incidence of preeclampsia, and also to explore the benefits of inclusion in the risk assessment and management system of preeclampsia for reducing the incidence of preeclampsia. 4. To evaluate the influence of the preeclampsia risk assessment and management model on the decrease of preeclampsia incidence, mortality and premature birth rate in pregnant women in Shanxi province. |
